# Clinical Course, Viral Load, and (Sub)Type of Influenza in Hospitalized Children in Southern Germany

**DOI:** 10.1002/jmv.71096

**Published:** 2026-08-11

**Authors:** Sarah Klemm, Clara Marlene Ihling, Julia Gsenger, Maximillian Stich, Johannes Pfeil, Silan Ünal, Marianne Wedde, Ralf Dürrwald, Paul Schnitzler, Julia Tabatabai

**Affiliations:** ^1^ Centre for Infectious Diseases, Virology, Medical Faculty Heidelberg Heidelberg University Heidelberg Germany; ^2^ Deutsches Zentrum für Infektionsforschung (DZIF), Partner Site Heidelberg Heidelberg Germany; ^3^ Children's Hospital, Deaconess Foundation Hospital Speyer Speyer Germany; ^4^ Dr. von Hauner Children's Hospital University Hospital of the LMU Munich Munich Germany; ^5^ Department of Paediatrics I Centre for Paediatric and Adolescent Medicine Medical Faculty Heidelberg Heidelberg University Heidelberg Germany; ^6^ Kinder‐ und Hausarztpraxis Schwaigern Germany; ^7^ Darmstädter Kinderkliniken Darmstadt Germany; ^8^ Robert Koch Institute, Unit 17: Influenza and Other Respiratory Viruses, National Reference Centre for Influenza Berlin Germany

**Keywords:** children, influenza virus, molecular epidemiology, phylogenetic analysis

## Abstract

This study aimed to assess the relationship between clinical course and influenza virus characteristics by quantifying viral load and performing phylogenetic analyses in hospitalized children. Children aged 0–3 years presenting with an acute respiratory tract infection (RTI) at the Childrens' Hospital Heidelberg/Germany were included and nasopharyngeal swabs were collected. Viral load was quantified by real‐time polymerase chain reaction and influenza virus (sub)type was identified by haemagglutinin gene sequencing. Among 1464 respiratory samples collected, 139 were positive for influenza virus, and 112 samples were selected for viral load analysis and sequencing. Influenza‐positive children were significantly older than influenza‐negative children (*p* < 0.001) and presented with symptoms of rhinitis (94.8%), cough (83.7%), and fever (54.3%). They presented most frequently with admission diagnoses of upper RTI (22.3%), febrile convulsion (18.7%), and pneumonia (15.8%). Children with high viral load were younger (*p* < 0.001), however clinical presentation was not associated with viral load levels. Comparing influenza A (*n* = 104/139) and B (*n* = 35/139) infections, no differences in demographic or clinical characteristics were identified. Children with influenza A(H1N1)pdm09 (*n* = 54/104) had pneumonia (*p* = 0.002), tachypnoea (*p* = 0.004), rales (*p* = 0.041), and retractions (*p* = 0.015) more frequently compared to influenza A(H3N2)‐infected children (*n* = 50/104). Phylogenetic analyses showed that the identified influenza virus strains were consistent with nationwide and European data in the corresponding seasons. Viral load and influenza type A compared to B did not correlate with a more severe course of disease; however, viral load was higher in younger children and influenza subtype A(H1N1)pdm09 was significantly associated with lower RTI.

## 1 Introduction

Influenza virus is an important pathogen of medically attended acute respiratory tract infection (RTI) in children and globally attributed—before the SARS‐CoV‐2 pandemic—to around 10% of respiratory hospitalizations in children below the age of 18 years worldwide [1].

Globally, there are an estimated 109.5 million influenza episodes in children under 5 years of age each year, including 10.1 million cases of acute lower RTI [2]. The distribution and increased use of polymerase chain reaction (PCR) analysis have effectively improved the sensitivity and speed of diagnosing influenza virus infection.

Through antigenic drift and shift, influenza strains escape the long‐term immunity of their hosts, causing annual epidemics and, at irregular intervals, pandemics. The annual influenza vaccination recommendations in Germany currently include people aged 60 and over, individuals aged 6 months and older with underlying diseases, pregnant women, and medical staff. Overall, the general influenza vaccination coverage in Germany is very low. Vaccination rates among children with underlying medical conditions were < 20% between 2009 and 2018 [3, 4]. Surveillance of influenza viruses allows early detection of variants not covered by the vaccine and new genotypes, and can help to prevent or contain epidemics and pandemics.

Influenza viruses are classified into subtypes based on the surface antigens haemagglutinin (HA) and neuraminidase (NA). In recent years, only influenza A viruses H3N2 and H1N1 have circulated as human‐pathogenic subtypes. Influenza B viruses are divided into two different lineages based on HA and NA, the Victoria and Yamagata lineages. Influenza viruses can be further classified into genetic groups and subgroups through sequencing of the HA gene. Phylogenetic trees can be used to trace the evolution of strains, with genetic clusters usually dominating for about 1 to 5 years [5]. Influenza A(H3N2) viruses change most rapidly, followed by influenza A(H1N1) viruses, whereas influenza B viruses change more slowly [5, 6]. Especially for influenza viruses, continuous molecular genetic surveillance of circulating strains in combination with clinical data is important. Quantifying viral load may also help to understand the interaction between the pathogen and the host and its association with disease severity. However, data on viral load, molecular characteristics, and the clinical course of influenza infection in children are limited [7].

This study aimed to investigate the association between clinical presentation and influenza virus characteristics in hospitalized children by quantifying viral load and performing molecular and phylogenetic analyses.

## 2 Methods

### 2.1 Patients and Clinical Samples

We conducted a monocentric study of children up to 3 years of age admitted with acute respiratory tract infection (RTI) at the emergency department of the Childrens' Hospital (Centre for Pediatric and Adolescent Medicine) at the Heidelberg University Hospital, Germany, during the months October–April of winter seasons 2014/2015–2018/2019. Patient records and information were anonymized and de‐identified prior to analysis. Upper respiratory tract infection (URTI) was defined as the presence of respiratory symptoms (e.g., cough, sneezing) without signs of lower respiratory tract disease. Lower respiratory tract infection (LRTI) was defined as a diagnosis of pneumonia, bronchiolitis, or bronchitis on admission or signs of lower respiratory tract disease (e.g., rales, retractions). Nasopharyngeal swabs were taken prior to admission and sent to the Virology Department for further analysis. Clinical data were collected using a standardized questionnaire and included admission diagnosis and clinical symptoms.

### 2.2 PCR Analysis and Influenza Typing

RNA was extracted from respiratory specimens using the QIAamp Viral RNA Mini Kit (Qiagen, Hilden, Germany) according to the manufacturer's protocol. Reverse transcription (RT), amplification, and detection of viral RNA were performed using the RealStar Influenza Screen & Type RT‐PCR Kit 4.0 (altona Diagnostics, Hamburg, Germany) on a LightCycler 480 Instrument II (Roche, Mannheim, Germany) according to the manufacturer's instructions. This assay distinguished influenza viruses A, A(H1N1)pdm09, and B. Samples and extracted RNA that tested positive were stored frozen at −20°C at the Clinical Virology Laboratory.

### 2.3 Analysis of Viral Load

Influenza virus RNA quantification standards from Vircell (Cologne, Germany) were serially diluted to generate standard curves for influenza viral load. A quantification standard with 1 × 10^6^ copies/mL was consecutively included in every run as a reference point to align the standard curve. Viral load values of patient samples were determined using the stored standard curves. Viral load results for infected children were divided into two groups, children with low viral load and children with high viral load, by determining a cut‐off according to the mean value in the histogram and then dichotomized for the overall analysis.

### 2.4 Sequencing of Influenza Virus Strains

Classification of influenza subtype was achieved by sequencing of the viral haemagglutinin (HA) gene according to in‐house protocols kindly provided by the Robert Koch Institute. Therefore, multi‐segment one‐step RT‐PCR was used to create and amplify complementary DNA fragments from viral RNA templates according to Zhou et al. [8, 9]. After successful amplification of the template RNA, nested‐PCR was performed to specifically amplify the HA gene using different sets of primers, resulting in partially overlapping fragments as previously described [10]. After purification of PCR products, they were sequenced at GATC Biotech (Cologne, Germany). Identified HA sequences of the collected samples were deposited in GenBank (https://www.ncbi.nlm.nih.gov/genbank/) and can be retrieved under the reference numbers OP847798‐OP847849 (Influenza A) as well as OP848016‐OP848028 (Influenza B).

### 2.5 Phylogenetic Analysis

The obtained single sequencing files were assembled and edited using SeqMan Pro Software of the Lasergene package (DNASTAR, Madison, Wisconsin, USA). MEGA 7.0.26 was used to translate nucleotide sequences to amino acid sequences and create an alignment using the Clustal W method [11]. This alignment allowed for identification of non‐synonymous substitutions relative to A/Texas/50/2012 (A(H3N2)), A/California/07/2009 (A(H1N1)pdm09), B/Malaysia/2506/2004 (B/Victoria), or B/Florida/4/2006 (B/Yamagata); silent nucleotide mutations which did not lead to amino acid substitutions were disregarded. Alignments of the sample collective were compared to reference sequences, which were chosen according to the seasonal WHO reports for September 2014–February 2019 and were obtained from the online data base Global Initiative on Sharing All Influenza Data (GISAID) [12, 13, 14]. The accession numbers of the sequences retrieved from GISAID are provided in the supporting material (Supplement 1). A phylogenetic tree for each influenza subtype, containing collected sample sequences as well as reference sequences was constructed using the maximum‐likelihood method with 1000 bootstraps, based on the Jones–Taylor–Thornton model with uniform rates and the use of all sites for gap or missing data treatment [15].

### 2.6 Statistical Analysis

Stata/SE 17.0 (StataCorp LLC, College Station, Texas, USA) was used for statistical analysis. For descriptive analysis, averages, ranges, and proportions were applied accordingly. For normally distributed data, continuous variables were described by their means and standard deviation. Student's *t*‐test, chi‐square test, and fisher's exact test were used for significance (*p* < 0.05) of differences in univariate analysis. Parameters significant in univariate analysis of viral load were further analyzed in logistic regression models adjusted for age, sex, and duration of symptoms prior to testing, as viral load is known to decrease during influenza infection.

### 2.7 Ethical Approval

The Ethical Committee of the Heidelberg University Hospital has approved this study (S‐547/2015). All patient data was pseudonymized, and there was no access to personal identifiers during data analysis. The Ethical Research Board of the University of Heidelberg exempted this retrospective analysis from any informed consent procedures, as only routinely obtained data and samples were used.

## 3 Results

### 3.1 Patient Characteristics and Epidemiology

**Figure 1:**
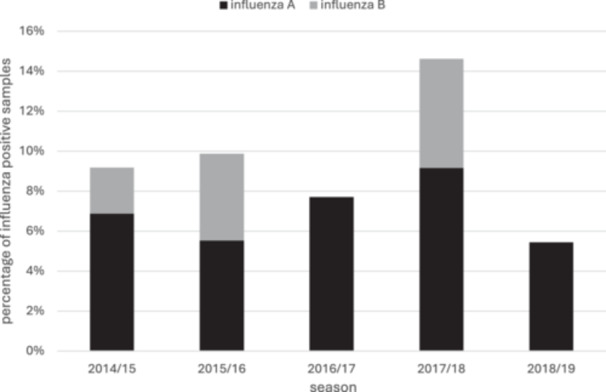
Distribution of influenza virus detection in children from seasons 2014/2015 to 2018/2019.

All children up to 3 years of age who presented to the emergency department of the Centre for Pediatric and Adolescent Medicine at Heidelberg University Hospital with signs of acute RTI were included in the study. In the winter seasons 2014/2015–2018/2019, a total of 1464 respiratory samples were obtained, of which 139 (9.5%) samples tested positive for the influenza virus by RT‐PCR. The proportion of influenza‐positive samples was highest in 2017/2018 (*n* = 51/349; 14.6%) and lowest in the 2018/2019 season (*n* = 16/294; 5.4%), see Figure 1. The demographic characteristics of the study population are shown in Table 1. The average age of all children was 1 year, influenza‐positive children were significantly older compared to influenza‐negative children (mean age 1.4 vs. 1.0 years; *p* < 0.001). The proportion of influenza‐positive children increased with age across the age groups (*p* < 0.001). However, as most tested hospitalized children were younger than 2 years (*n* = 1226/1464; 83.7%), most influenza‐positive children in this study were also younger than 2 years (*n* = 100/139; 71.9%).

**Table 1:** Demographic characteristics of the study population.

	Total	Influenza positive	Influenza negative	*p* value	Influenza A	Influenza B	*p* value
	*n* = 1464	*n* = 139	*n* = 1325		*n* = 104	*n* = 35	
Sex, *n* (%)				0.713^b^			0.886^b^
Male	801 (54.7%)	74 (53.2%)	727 (54.9%)		55 (52.9%)	19 (54.3%)	
Female	663 (45.3%)	65 (46.8%)	598 (45.1%)		49 (47.1%)	16 (45.7%)	
Age in years, mean (SD)	1.0 (±1.0)	1.4 (±1.1)	1.0 (±1.0)	<0.001***^a^	1.5 (±1.1)	1.2 (±0.9)	0.221^a^
Age groups, *n* (%)				<0.001***^b^			0.273^b^
< 6 months	641 (43.8%)	38 (27.3%)	603 (45.5%)		28 (26.9%)	10 (28.6%)	
6 months–<1 year	232 (15.9%)	22 (15.8%)	210 (15.9%)		16 (15.4%)	6 (17.1%)	
1 year–<2 years	353 (24.1%)	40 (28.8%)	313 (23.6%)		26 (25.0%)	14 (40.0%)	
2 years–<3 years	153 (10.5%)	25 (18.0%)	128 (9.7%)		22 (21.2%)	3 (8.6%)	
3 years–<4 years	85 (5.8%)	14 (10.1%)	71 (5.4%)		12 (11.5%)	2 (5.7%)	

Abbreviations: kg, kilogram; SD, standard deviation.

Significant differences were tested using

^a^ Student's *t*‐test or

^b^ Pearson's χ^2^‐test.

### 3.2 Admission Diagnosis and Clinical Presentation

Overall, the most frequent admission diagnoses were bronchitis and bronchiolitis (25.9% and 21.4%; Table 2). These diagnoses were significantly more frequent in influenza‐negative children (*p* < 0.001). Among influenza‐positive children, the most common admission diagnoses were URTI (22.3%) and febrile convulsion (18.7%) and were significantly more frequent in influenza‐positive compared to influenza‐negative children (*p* = 0.015 and *p* < 0.001). Most children had rhinitis (89.3%) and cough (87.1%), see Table 3. More than half of the influenza‐positive children had fever (54.3%) and a higher mean body temperature (38.5°C vs. 38.0°C). Rhinitis and fever were significantly more frequent in influenza‐positive children (*p* = 0.028 and *p* < 0.001).

**Table 2:** Admission diagnosis in the study population.

Admission diagnosis	Total	Influenza positive	Influenza negative	*p* value	Influenza A	Influenza B	*p* value
	*n* = 1464	*n* = 139	*n* = 1325		*n* = 104	*n* = 35	
Respiratory							
URTI, *n* (%)	223 (15.2%)	31 (22.3%)	192 (14.5%)	0.015*^a^	25 (24.0%)	6 (17.1%)	0.397^a^
Rhinitis with difficulties to feed, *n* (%)	27 (1.8%)	3 (2.2%)	24 (1.8%)	0.737^b^	1 (1.0%)	2 (5.7%)	0.156^b^
Croup, *n* (%)	11 (0.8%)	3 (2.2%)	8 (0.6%)	0.078^b^	2 (1.9%)	1 (2.9%)	1.000^b^
Otitis media, *n* (%)	9 (0.6%)	0 (0%)	9 (0.7%)	1.000^b^	0 (0%)	0 (0%)	
Pneumonia, *n* (%)	190 (13.0%)	22 (15.8%)	168 (12.7%)	0.293^a^	16 (15.4%)	6 (17.1%)	0.805^a^
Bronchiolitis, *n* (%)	313 (21.4%)	12 (8.6%)	301 (22.7%)	< 0.001***^a^	11 (10.6%)	1 (2.9%)	0.295^b^
Bronchitis, *n* (%)	379 (25.9%)	18 (13.0%)	361 (27.3%)	< 0.001***^a^	9 (8.7%)	9 (25.7%)	0.017*^b^
Non‐respiratory							
Febrile convulsion, *n* (%)	119 (8.1%)	26 (18.7%)	93 (7.0%)	< 0.001***^a^	20 (19.2%)	6 (17.1%)	0.784^a^
Fever of unknown origin, *n* (%)	39 (2.7%)	7 (5.0%)	32 (2.4%)	0.089^b^	6 (5.8%)	1 (2.9%)	0.679^b^
Bad general condition, *n* (%)	16 (1.1%)	1 (0.7%)	15 (1.1%)	1.000^b^	1 (1.0%)	0 (%)	1.000^b^
Gastroenteritis, *n* (%)	14 (1.0%)	4 (2.9%)	10 (0.8%)	0.037*^b^	3 (2.9%)	1 (2.9%)	1.000^b^
Others, *n* (%)	124 (8.5%)	12 (8.6%)	112 (8.5%)	0.942^a^	10 (9.6%)	2 (5.7%)	0.730^b^
URTI, *n* (%)	496 (33.9%)	78 (56.1%)	418 (31.6%)	< 0.001***^a^	61 (58.7%)	17 (48.6%)	0.298^a^
LRTI, *n* (%)	968 (66.1%)	61 (43.9%)	907 (68.5%)		43 (41.4%)	18 (51.4%)	

Abbreviations: LRTI, lower respiratory tract infection; URTI, upper respiratory tract infection.

Significant differences were tested using

^a^ Pearson's χ^2^‐test or

^b^ Fisher's exact‐test.

**Table 3:** Clinical characteristics of the study population.

	Total	Influenza positive	Influenza negative	*p* value	Influenza A	Influenza B	*p* value
	*n* = 1464	*n* = 139	*n* = 1325		*n* = 104	*n* = 35	
Symptoms							
Fever, % (*n*/*N*)	34.5% (471/1366)	54.3% (69/127)	32.5% (402/1239)	<0.001***^b^	56.4% (53/94)	48.5% (16/33)	0.433^b^
Rhinitis, % (*n*/*N*)	89.3% (1279/1433)	94.8% (128/135)	88.7% (1151/1298)	0.028*^b^	95.0% (95/100)	94.3% (33/35)	1.000^c^
Hoarseness, % (*n*/*N*)	18.4% (226/1230)	10.1% (11/109)	19.2% (215/1121)	0.019*^b^	6.3% (5/80)	20.7% (6/29)	0.065^c^
Cough, % (*n*/*N*)	87.1% (1251/1437)	83.7% (113/135)	87.4% (1138/1302)	0.223^b^	82.2% (83/101)	88.2% (30/34)	0.408^b^
Nasal flaring, % (*n*/*N*)	13.6% (190/1395)	4.7% (6/129)	14.5% (184/1266)	0.002**^b^	4.2% (4/95)	5.9% (2/34)	0.654^c^
Retractions, % (*n*/*N*)	42.1% (594/1411)	25.2% (33/131)	43.8% (561/1280)	< 0.001***^b^	22.7% (22/97)	32.4% (11/34)	0.264^b^
Tachypnoea, % (*n*/*N*)	49.0% (696/1421)	33.3% (45/135)	50.6% (651/1286)	< 0.001***^b^	30.7% (31/101)	41.2% (14/34)	0.262^b^
Apnea, % (*n*/*N*)	4.3% (58/1366)	1.6% (2/128)	4.5% (56/1238)	0.114^b^	2.1% (2/95)	0% (0/33)	1.000^c^
Cyanosis, % (*n*/*N*)	7.8% (109/1395)	7.7% (10/130)	7.8% (99/1265)	0.957^b^	6.2% (6/97)	12.1% (4/33)	0.273^c^
Stridor, % (*n*/*N*)	10.4% (145/1389)	6.8% (9/133)	10.8% (136/1256)	0.145^b^	5.1% (5/98)	11.4% (4/35)	0.242^c^
Wheezing, % (*n*/*N*)	36.6% (521/1423)	18.5% (25/135)	38.5% (496/1288)	< 0.001***^b^	14.0% (14/100)	31.4% (11/35)	0.022*^b^
Rales, % (*n*/*N*)	39.8% (567/1426)	31.3% (42/134)	40.6% (525/1292)	0.036*^b^	29.0% (29/100)	38.2% (13/34)	0.316^b^
Other clinical characteristics							
Temperature at admission in °C, mean (SD)†	38.0 (±1.0)	38.5 (±1.0)	38.0 (±1.0)	< 0.001***^a^	38.6 (±1.0)	38.3 (±0.8)	0.153^a^
Duration of symptoms prior to admission in days, mean (SD)‡	4.5 (±5.5)	4.5 (±4.8)	4.5 (±5.6)	0.987^a^	4.6 (±4.9)	4.4 (±4.6)	0.892^a^
Hospital stay in days, mean (SD)	4.5 (±7.1)	3.9 (±3.6)	4.6 (±7.4)	0.278^a^	3.9 (±3.9)	4.0 (±2.6)	0.891^a^

*Note:* Significant differences were tested using Student's *t*‐test, Pearson's χ^2^‐test or Fisher's exact‐test.

Abbreviation: SD, standard deviation.

^†^ Information available for *n* = 1301.

^‡^ Information available for *n* = 1366.

^a^ Student's *t*‐test,

^b^ Pearson's χ^2^‐test, or

^c^ Fisher's exact‐test.

### 3.3 Viral Load

Viral load was categorized as low viral load (≤ 10^7^ copies/mL) and high viral load (> 10^7^ copies/mL). In univariate analysis, there was no association between viral load and sex (*p* = 0.322). When considering age, it is noticeable that children with high viral load were on average younger (1.1 years) than those with low viral load (1.8 years; *p* < 0.001). In a logistic regression model adjusted for age, sex, and duration of symptoms, age remained significantly associated with viral load. Except for pneumonia, which was more frequent in children with low viral load, no association between other admission diagnoses and viral load levels was observed in univariate analysis (*p* > 0.05). Adjusted for age, sex, and duration of symptoms, pneumonia was no longer significant in the logistic regression. There was also no discernible association between various symptoms and viral load, nor between URTI or LRTI and viral load (*p* > 0.05). Similarly, there was no significant difference in viral load for body temperature as well as for length of hospital stay (*p* > 0.05). The mean duration of symptoms before admission was 4.8 days and shorter in children with high viral load (3.5 days) compared to children with low viral load, who had symptoms for 6.2 days (*p* = 0.008). The association between shorter duration of symptoms and a higher viral load remained significant in multivariate analysis adjusted for age and sex (see also Supplement 2).

### 3.4 Influenza (Sub)Types

Of the 139 influenza‐positive samples, 104 were positive for influenza A and 35 for influenza B. Comparing influenza types A and B, there was no significant difference in age or sex (Table 1). When considering admission diagnoses, bronchitis was more frequent in children with influenza B than in those with influenza A (*p* = 0.017); all other admission diagnoses showed no difference (Table 2). There was no association between URTI or LRTI and influenza types, as well as different symptoms and influenza types, except for wheezing, which was more common in influenza B‐infected children (*p* = 0.022). No difference was observed in the average duration of symptoms or duration of hospitalization (Table 3). Comparing influenza A subtypes, A(H1N1)pdm09 (*n* = 54/104) and A(H3N2) (*n* = 50/104), there was also no association between subtype and demographics. When considering admission diagnoses, it is noticeable that pneumonia was more frequent in children with influenza A(H1N1)pdm01 than in those with A(H3N2) (*p* = 0.002), but all other admission diagnoses showed no difference between the subtypes. Consistent with this finding, children with A(H1N1)pdm09 more frequently presented with retractions (*p* = 0.015), tachypnoea (*p* = 0.004), and rales (*p* = 0.041). No difference between URTI or LRTI and influenza A subtypes was observed.

### 3.5 Phylogenetic Analysis

**Figure 3:**
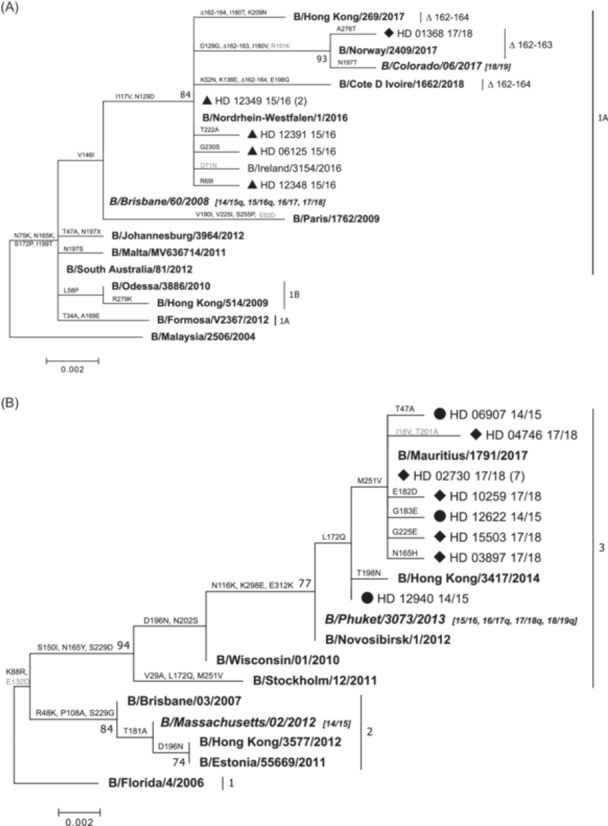
Phylogenetic analysis of influenza virus B/Victoria (A) and influenza virus B/Yamagata (B) haemagglutinin gene. Phylogenetic trees from amino acid sequences of influenza virus strains were constructed with maximum likelihood method and Jones‐Taylor‐Thornton‐model with 1000 bootstrap replicates using MEGA 7.0 software. Influenza virus strains from this study in Heidelberg, Germany are indicated by the abbreviation “HD” followed by the patient number and year of isolation (●2014/2015, ▲2015/2016, ■2016/2017, ♦2017/2018, ▼2018/2019). The number in brackets indicates the number of additional samples with an identical amino acid sequence. **Reference strains** were retrieved from GISAID and **are indicated in bold** and by influenza type/place of isolation/isolate number/year of isolation. ***Vaccine strains are indicated in bold and italics***, and the years in which they were used are indicated in square brackets; strains with q only were used in quadrivalent vaccines. Assignment to genetic groups is indicated on the right with the respective name of the group. Bootstrap values greater than 70% are indicated at branch nodes. The scale bar represents the number of amino acid substitutions per site. Amino acid substitutions are shown in comparison to B/Malaysia/2506/2004 (A) and B/Florida/4/2006 (B). Amino acid substitutions in the HA1 subunit are indicated in black, in the HA2 subunit in gray.

Sequencing of the HA gene of influenza viruses was successfully performed in 112 samples and phylogenetic trees were constructed. In total, sequences of 48 A(H1N1)pdm09, 42 A(H3N2), and 22 influenza B strains were identified. In winter 2014/2015, A(H3N2) viruses of groups 3 C.3, 3 C.3b and 3 C.2a were found in the patient collective (Figure 2A). No A(H3N2) viruses were identified in the 2015/2016 season, whereas in the 2016/2017 season, A(H3N2) was the dominant subtype. All influenza viruses in 2016/2017 belonged to group 3 C.2a and its subgroups, mainly 3 C.2a1. In winter 2017/2018, only one A(H3N2) sample was successfully sequenced, which belonged to group 3 C.2a1b. In the 2018/2019 season, viruses of groups 3 C.2a1b and 3 C.3a circulated. All sequenced influenza A(H1N1)pdm09 viruses from the 2014/2015 to 2018/2019 seasons belonged to genetic group 6B (Figure 2B). In the 2015/2016 season, only A(H1N1)pdm09 viruses of subgroup 6B.1 were identified. The only A(H1N1)pdm09‐positive sample from winter 2016/2017 also belonged to subgroup 6B.1. The sequenced viruses from the 2017/2018 and 2018/2019 seasons fell into subgroup 6B.1 A. Victoria lineage influenza B viruses were only identified in the 2015/2016 and 2017/2018 seasons, and all belonged to group 1A (Figure 3A). Influenza B viruses of the Yamagata lineage were identified only in the 2014/2015 and 2017/2018 seasons, belonging to group 3 (Figure 3B).

Figure 2Phylogenetic analysis of influenza virus A(H3N2) (A) and influenza virus A(H1N1)pdm09 (B) haemagglutinin gene. Phylogenetic trees from amino acid sequences of influenza virus strains were constructed with maximum likelihood method and Jones–Taylor–Thornton‐model with 1000 bootstrap replicates using MEGA 7.0 software. Influenza virus strains from this study in Heidelberg, Germany are indicated by the abbreviation “HD” followed by the patient number and year of isolation (●2014/2015, ▲2015/2016, ■2016/2017, ♦2017/2018, ▼2018/2019). The number in brackets indicates the number of additional samples with an identical amino acid sequence. **Reference strains** were retrieved from GISAID and **are indicated in bold** and by influenza type/place of isolation/isolate number/year of isolation. ***Vaccine strains are indicated in bold and italics***, and the years in which they were used are indicated in square brackets; strains with q only were used in quadrivalent vaccines. Assignment to genetic groups is indicated on the right with the respective name of the group. Bootstrap values greater than 70% are indicated at branch nodes. The scale bar represents the number of amino acid substitutions per site. Amino acid substitutions are shown in comparison to A/Texas/50/2012 (A) and A/California/07/2009 (B). Amino acid substitutions in the HA1 subunit are indicated in black, in the HA2 subunit in gray.

**Figure:**
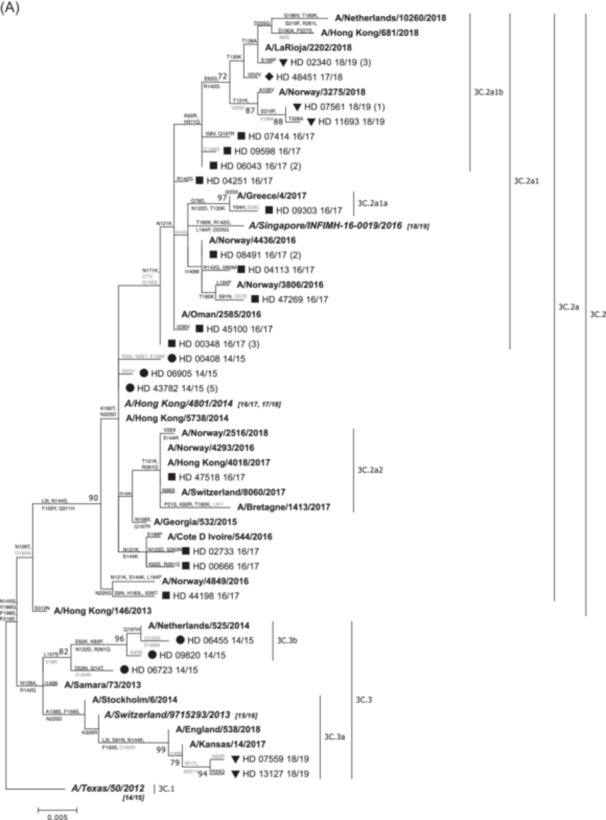

**Figure:**
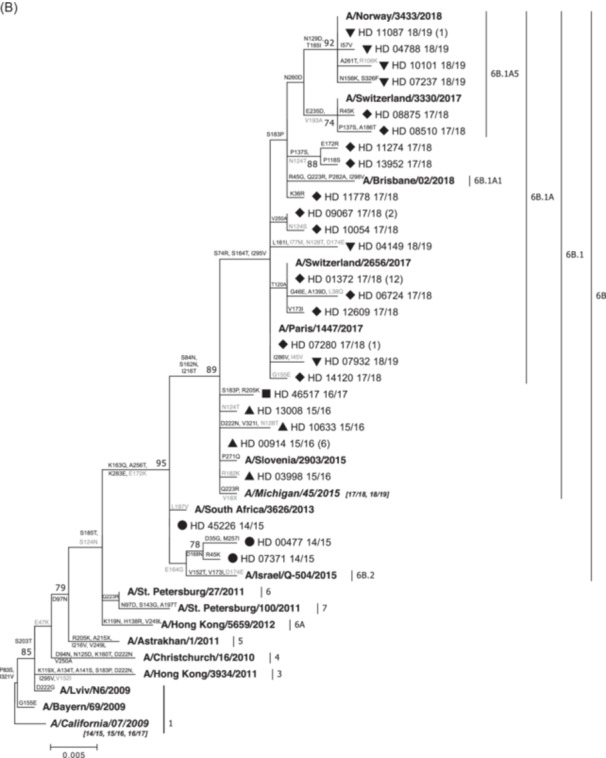

## 4 Discussion

This study investigated the clinical presentation of influenza virus infection in hospitalized children according to viral load and influenza (sub)type, as well as the molecular epidemiology of influenza viruses during the 2014/2015–2018/2019 winter seasons. Influenza viruses were identified in 9.5% of all admitted children aged 0–3 years with signs of acute RTI. Influenza‐positive children were older and more often admitted with a diagnosis of URTI than influenza‐negative children. An association between viral load and clinical disease severity could not be demonstrated. Children with high viral load were on average younger. This could also be confirmed in a logistic regression model adjusted for duration of symptoms and sex. There was no difference between influenza virus A and B with respect to demographic characteristics and clinical symptoms. Comparing influenza A subtypes, there was no difference in demographics, and clinical presentation was similar, with a potentially more severe course of A(H1N1)pdm09 infections.

The clinical and demographic features of our study are comparable to those reported in the literature. In line with Lakhan et al., the majority of influenza‐positive children were under 2 years of age [16]. Younger children are at higher risk of hospitalization and a severe course of influenza infection [1, 17]. In this study, influenza‐positive children were older than influenza‐negative children. Moreover, influenza‐positive infants and toddlers who presented to our emergency department were also significantly older than children who tested positive for other respiratory viruses, such as respiratory syncytial virus (RSV) as previously described elsewhere [18, 19, 20, 21]. RSV is the most important causative agent of acute respiratory infections in hospitalized infants and young children [22]. The hospitalization rate for RSV is 16 times higher than for influenza in children under 1 year of age [23].

The most common diagnoses in influenza‐positive children were URTI, febrile convulsion, and pneumonia. These admission diagnoses reflect the typical clinical presentation and common complications of influenza in children [24, 25]. The most common symptoms among influenza‐positive children were rhinitis, cough, and fever. This is consistent with findings from other studies [16, 26].

In our study population, children with high viral load were significantly younger. In influenza, viral shedding and accordingly viral load correlate with the onset of symptoms and peak around symptom onset to 3 days after [27, 28]. The association between a shorter duration of symptoms and higher viral load is consistent with the natural course of influenza infection. Influenza viral replication is highest during the first few days after symptom onset and declines thereafter as the host immune response develops; therefore, children presenting early in the disease course are expected to have higher viral loads than those presenting later [7, 29].

The duration of symptoms before admission was shorter in children with high viral load than in those with low viral load; however, the association of young age and high viral loads remained in logistic regression adjusted for duration of symptoms. Granados et al. also describe higher viral loads in children under 1 year of age infected with influenza A(H3N2) or B compared to children aged 1–4 years [30]. Another study also showed higher viral loads in young children [31]. Higher viral loads in younger children could be a result of a primary infection and/or due to the maturity of the immune system [32]. However, many other studies did not find a difference in viral load depending on age [33, 34, 35].

In our study, no association between viral load and admission diagnoses or symptoms, or length of hospital stay was found. Li et al. described higher A(H1N1)pdm09 viral loads in patients with pneumonia than in patients with URTI or bronchitis [33]. Granados et al. found higher viral loads in hospitalized patients with influenza B than in outpatients [30].

Influenza A‐ and B‐infected children presented a very similar clinical picture. Except for bronchitis and wheezing, which were more common in children with influenza B, there was no difference between influenza A and B in terms of demographic characteristics, admission diagnoses, symptoms, and length of hospital stay. Most studies also found no differences in clinical presentation, duration of illness, and disease severity between the two types [25, 36]. However, when comparing influenza A subtypes, A(H1N1)pdm09‐positive children were more frequently diagnosed with pneumonia on admission than A(H3N2)‐positive children and more often presented with typical symptoms of pneumonia, such as tachypnoea, retractions, and pulmonary rales. The length of hospital stay did not differ between the influenza A subtypes. According to Caini et al., influenza A subtype does not determine the clinical picture of infection, but A(H1N1)pdm09 may cause more complications and a more severe course of disease, as also described by others [37, 38].

Molecular epidemiology attempts to establish a relationship among the pattern and course of disease, the genotype of the pathogen, and characteristics of the host, and thus to identify risk factors and correlate pathogenicity with genotype. In this study, influenza A was the dominant type in all seasons. The dominant subtype and the proportion of co‐circulating influenza B viruses and their dominant lineage varied from season to season. In the period 2014/2015–2018/2019, influenza viruses of subtype A(H3N2) evolved from genetic groups 3C.2a and 3C.3 towards 3C.3a and especially 3C.2a1b. Among the influenza viruses, A(H3N2) viruses change most rapidly, as reflected by the constant evolution of new subgroups and frequent subsequent changes in vaccine strains [6]. The HA genes of the A(H1N1)pdm09 viruses of the 2014/2015–2018/2019 winter seasons all belonged to genetic group 6B and its subgroups. During the study period, an evolution of the A(H1N1)pdm09 viruses from genetic group 6B to group 6B.1A5 was observed. Influenza B viruses change more slowly, do not circulate every season, and usually occur to a lesser extent than influenza A viruses [5]. B/Victoria was the dominant influenza B lineage in Germany only in the 2015/2016 season [39]. In the 2017/2018 season only a very small number of B/Victoria viruses circulated in general, and in our patient population only one strain was identified. In the 2014/2015 and 2017/2018 seasons, B/Yamagata was the dominant lineage. All B/Yamagata viruses from this study belonged to group 3. The amino acid substitutions in influenza strains detected in this study, as well as their distribution among the different genetic (sub)groups, corresponded to those described by the Robert Koch Institute for Germany and the Crick Institute for Europe [39, 40].

This study has several limitations. As only children with signs of acute RTI were included, influenza positive children with fever only or otherwise atypical presentation without respiratory symptoms may not have been included. In addition, the study was limited to hospitalized children and therefore focused on a more severe clinical presentation.

Information on influenza vaccination status was not available for the study cohort. However, given the restrictive vaccination recommendations during the study period and the low vaccination coverage among eligible children in Germany, the number of vaccinated children in our cohort was likely very small, precluding meaningful analyses according to vaccination status.

In conclusion, younger children had higher influenza viral loads, and viral load was higher the shorter the duration of symptoms was. No other associations were found between viral load and clinical characteristics. Clinical presentation of influenza infections of both types, A and B, was similar. In contrast, the comparison of influenza A subtypes suggests a possibly more severe course of A(H1N1)pdm09 infections due to the more frequent occurrence of pneumonia and respiratory insufficiency. Phylogenetic analysis showed a continuous evolution of genotypes, with the appearance of new amino acid substitutions and genetic groups. Monitoring the molecular evolution of influenza viruses is crucial to detect the emergence of new variants and to continuously improve vaccination and treatment strategies.

## Author Contributions

Conception or design of the work: Sarah Klemm, Paul Schnitzler, Julia Tabatabai. Data collection: Sarah Klemm, Clara Marlene Ihling, Julia Gsenger, Maximillian Stich, Johannes Pfeil, Paul Schnitzler, Julia Tabatabai. Study protocols provided by the Robert Koch Institute: Marianne Wedde, Ralf Dürrwald. Data analysis and interpretation: Sarah Klemm, SK, Paul Schnitzler, Julia Tabatabai. Drafting the article: Sarah Klemm, Paul Schnitzler, Julia Tabatabai. Critical revision of the article: Sarah Klemm, Clara Marlene Ihling, Julia Gsenger, Maximillian Stich, Johannes Pfeil, Silan Ünal, Marianne Wedde, Ralf Dürrwald, Paul Schnitzler, Julia Tabatabai. Final approval of the version to be published: Sarah Klemm, Clara Marlene Ihling, Julia Gsenger, Maximillian Stich, Johannes Pfeil, Silan Ünal, Marianne Wedde, Ralf Dürrwald, Paul Schnitzler, Julia Tabatabai.

## Conflicts of Interest

The authors declare no conflicts of interest.

## Supporting information


**Supplement 1:** Reference sequences of the haemagglutinin gene retrieved from GISAID (Global Initiative on Sharing All Influenza Data). Reference sequences for the alignment with our sample collection were chosen according to the seasonal WHO reports of the years September 2014–February 2019 and added via the online database Global Initiative on Sharing All Influenza Data (GISAID). The accession numbers of all selected reference sequences are listed in a table.

**Supplement 2:** Logistic regression of viral load with duration of symptoms adjusted for age and sex. Parameters significant in univariate analysis of viral load were further analysed in logistic regression models adjusted for age, sex, and duration of symptoms prior to testing, as viral load is known to decrease during influenza infection. The table lists the odds ratio (OR), the 95% confidence interval (CI), and *p*‐values for each variable as well as for the overall model.

## Data Availability

The data that support the findings of this study are openly available in Genbank at http://www.ncbi.nlm.nih.gov/genbank, reference number OP847798‐OP847849; OP848016‐OP848028.
