# Supplementary material for: Clinical Course, Viral Load, and (Sub)Type of Influenza in Hospitalized Children in Southern Germany
Source: J Med Virol. 2026 Aug 11;98(8):e71096. doi: 10.1002/jmv.71096 (PMC13459153; doi:10.1002/jmv.71096)
Supplement: Supplementary file 1 [file JMV-98-e71096-s001.pdf]

Supplement 2: Logistic regression of viral load with duration of symptoms adjusted for age and sex.

LR: Viral load	OR	95% CI	p-value	overall p-value
Duration of symptoms (days)	0.88	0.79, 0.99	0.03	0.01
Age (years)	0.57	0.37, 0.87	0.01	
Sex (male)	1.77	0.74, 4.19	0.20	

LR: logistic regression; OR: odds ratio; CI: confidence intervall
