# Supplementary material for: Clinical Course, Viral Load, and (Sub)Type of Influenza in Hospitalized Children in Southern Germany
Source: J Med Virol. 2026 Aug 11;98(8):e71096. doi: 10.1002/jmv.71096 (PMC13459153; doi:10.1002/jmv.71096)
Supplement: Supplementary file 2 [file JMV-98-e71096-s002.pdf]

Supplement 1 Reference sequences of the haemagglutinin gene retrieved from GISAID (Global Initiative on Sharing All Influenza Data)

virus name	subtype/lineage	accession number	isolate ID	collection date	originating laboratory	submitting laboratory
A/California/07/2009	A(H1N1)pdm09	EPI516535	EPI_ISL_159428	09.04.2009	Naval Health Research Center U.S. Navy San Diego, United States	Centers for Disease Control and Prevention Atlanta, United States
A/Bayern/69/2009	A(H1N1)pdm09	EPI253705	EPI_ISL_73686	2009	Robert Koch Institute Nationales Referenzzentrum für Influenza Berlin, Germany	Robert Koch Institute Nationales Referenzzentrum für Influenza Berlin, Germany
A/Lviv/N6/2009	A(H1N1)pdm09	EPI215957	EPI_ISL_62012	27.10.2009	Ministry of Health of Ukraine Kiev, Ukraine	National Institute for Medical Research London, United Kingdom
A/Christchurch/16/2010	A(H1N1)pdm09	EPI280344	EPI_ISL_79722	12.07.2010	WHO Collaborating Centre for Reference and Research on Influenza Melbourne, Australia	Centers for Disease Control and Prevention Atlanta, United States
A/Hong Kong/3934/2011	A(H1N1)pdm09	EPI326206	EPI_ISL_93746	29.03.2011	Government Virus Unit Kowloon, Hong Kong (SAR)	National Institute for Medical Research London, United Kingdom
A/Astrakhan/1/2011	A(H1N1)pdm09	EPI319590	EPI_ISL_90787	28.02.2011	WHO National Influenza Centre Russian Federation St. Petersburg, Russian Federation	National Institute for Medical Research London, United Kingdom
A/St. Petersburg/100/2011	A(H1N1)pdm09	EPI320141	EPI_ISL_90954	14.03.2011	Russian Academy of Medical Sciences St. Petersburg, Russian Federation	Centers for Disease Control and Prevention Atlanta, United States
A/St. Petersburg/27/2011	A(H1N1)pdm09	EPI319527	EPI_ISL_90760	14.02.2011	WHO National Influenza Centre Russian Federation St. Petersburg, Russian Federation	National Institute for Medical Research London, United Kingdom
A/Hong Kong/5659/2012	A(H1N1)pdm09	EPI390473	EPI_ISL_127652	21.05.2012	Government Virus Unit Kowloon, Hong Kong (SAR)	National Institute for Medical Research London, United Kingdom
A/South Africa/3626/2013	A(H1N1)pdm09	EPI466626	EPI_ISL_145447	06.06.2013	National Institute for Communicable Disease Johannesburg, South Africa	National Institute for Medical Research London, United Kingdom

A/Israel/Q-504/2015	A(H1N1)pdm09	EPI697729	EPI_ISL_207902	15.12.2015	Central Virology Laboratory Israel (NIC)<br>Tel Hashomer, Israel	Crick Worldwide Influenza Centre<br>London, United Kingdom
A/Slovenia/2903/2015	A(H1N1)pdm09	EPI687827	EPI_ISL_206099	26.10.2015	Laboratory for Virology, National Institute of Public Health<br>Ljubljana, Slovenia	Crick Worldwide Influenza Centre<br>London, United Kingdom
A/Paris/1447/2017	A(H1N1)pdm09	EPI1142011	EPI_ISL_291668	20.10.2017	Institut Pasteur<br>Paris, France	Crick Worldwide Influenza Centre<br>London, United Kingdom
A/Switzerland/2656/2017	A(H1N1)pdm09	EPI1153818	EPI_ISL_294119	21.12.2017	Swiss National Reference Centre for Influenza<br>Geneva, Switzerland	Crick Worldwide Influenza Centre<br>London, United Kingdom
A/Switzerland/3330/2017	A(H1N1)pdm09	EPI1153822	EPI_ISL_294121	20.12.2017	Swiss National Reference Centre for Influenza<br>Geneva, Switzerland	Crick Worldwide Influenza Centre<br>London, United Kingdom
A/Brisbane/02/2018	A(H1N1)pdm09	EPI1312566	EPI_ISL_330190	04.01.2018	WHO Collaborating Centre for Reference and Research on Influenza<br>Melbourne, Australia	Centers for Disease Control and Prevention<br>Atlanta, United States
A/Norway/3433/2018	A(H1N1)pdm09	EPI1328929	EPI_ISL_332840	30.10.2018	WHO National Influenza Centre<br>Oslo, Norway	Crick Worldwide Influenza Centre<br>London, United Kingdom
A/Texas/50/2012	A(H3N2)	EPI397876	EPI_ISL_129744	15.04.2012	Texas Department of State Health Services-Laboratory Services<br>Austin, United States	Centers for Disease Control and Prevention<br>Atlanta, United States
A/Samara/73/2013	A(H3N2)	EPI460558	EPI_ISL_143568	12.03.2013	WHO National Influenza Centre Russian Federation<br>St. Petersburg, Russian Federation	National Institute for Medical Research<br>London, United Kingdom
A/Switzerland/9715293/2013	A(H3N2)	EPI530687	EPI_ISL_162149	06.12.2013	Swiss National Reference Centre for Influenza<br>Geneva, Switzerland	National Institute for Medical Research<br>London, United Kingdom
A/Hong Kong/146/2013	A(H3N2)	EPI426061	EPI_ISL_136375	11.01.2013	Government Virus Unit<br>Kowloon, Hong Kong (SAR)	National Institute for Medical Research<br>London, United Kingdom
A/Stockholm/6/2014	A(H3N2)	EPI530677	EPI_ISL_162144	06.02.2014	Public Health Agency of Sweden<br>Solna, Sweden	National Institute for Medical Research<br>London, United Kingdom

A/Netherlands/525/2014	A(H3N2)	EPI574644	EPI_ISL_175051	17.12.2014	National Institute for Public Health and the Environment (RIVM)<br>Bilthoven, Netherlands	National Institute for Medical Research<br>London, United Kingdom
A/Hong Kong/5738/2014	A(H3N2)	EPI539806	EPI_ISL_165629	30.04.2014	Government Virus Unit<br>Kowloon, Hong Kong (SAR)	National Institute for Medical Research<br>London, United Kingdom
A/Hong Kong/4801/2014	A(H3N2)	EPI539576	EPI_ISL_165554	26.02.2014	Government Virus Unit<br>Kowloon, Hong Kong (SAR)	National Institute for Medical Research<br>London, United Kingdom
A/Georgia/532/2015	A(H3N2)	EPI641435	EPI_ISL_195324	09.03.2015	nicht verfügbar	Autoren:<br>Machablishvili A, Gavashelidze M, Sabadze L, Jashiashvili T,
A/Norway/3806/2016	A(H3N2)	EPI922445	EPI_ISL_249683	13.06.2016	WHO National Influenza Centre<br>Oslo, Norway	Crick Worldwide Influenza Centre<br>London, United Kingdom
A/Norway/4293/2016	A(H3N2)	EPI868793	EPI_ISL_239775	22.10.2016	WHO National Influenza Centre<br>Oslo, Norway	Crick Worldwide Influenza Centre<br>London, United Kingdom
A/Norway/4436/2016	A(H3N2)	EPI868813	EPI_ISL_239785	03.11.2016	WHO National Influenza Centre<br>Oslo, Norway	Crick Worldwide Influenza Centre<br>London, United Kingdom
A/Norway/4849/2016	A(H3N2)	EPI1140343	EPI_ISL_291279	2016	Crick Worldwide Influenza Centre<br>London, United Kingdom	WHO Collaborating Centre for Reference and Research on Influenza<br>Melbourne, Australia
A/Cote d'Ivoire/544/2016	A(H3N2)	EPI781596	EPI_ISL_226075	06.04.2016	Pasteur Institut of Côte d'Ivoire<br>Abidjan, Cote d'Ivoire	Crick Worldwide Influenza Centre<br>London, United Kingdom
A/Oman/2585/2016	A(H3N2)	EPI769531	EPI_ISL_223655	04.03.2016	Central Public Health Laboratory, Ministry of Health<br>Muscat, Oman	Crick Worldwide Influenza Centre<br>London, United Kingdom
A/Singapore/INFIMH-16-0019/2016	A(H3N2)	EPI1381189	EPI_ISL_344420	14.06.2016	WHO Chinese National Influenza Center<br>Beijing, China	WHO Chinese National Influenza Center<br>Beijing, China
A/Kansas/14/2017	A(H3N2)	EPI1146345	EPI_ISL_292575	14.12.2017	Kansas Department of Health and Environment<br>Topeka, United States	Centers for Disease Control and Prevention<br>Atlanta, United States
A/Bretagne/1413/2017	A(H3N2)	EPI1145228	EPI_ISL_292377	09.10.2017	Institut Pasteur<br>Paris, France	Crick Worldwide Influenza Centre<br>London, United Kingdom

A/Switzerland/8060/2017	A(H3N2)	EPI1154878	EPI_ISL_294252	21.12.2017	Swiss National Reference Centre for Influenza<br>Geneva, Switzerland	Crick Worldwide Influenza Centre<br>London, United Kingdom
A/Hong Kong/4018/2017	A(H3N2)	EPI539576	EPI_ISL_165554	26.02.2014	Government Virus Unit<br>Kowloon, Hong Kong (SAR)	National Institute for Medical Research<br>London, United Kingdom
A/Greece/4/2017	A(H3N2)	EPI967219	EPI_ISL_257379	02.01.2017	Aristotelian University of Thessaloniki<br>Thessaloniki, Greece	Crick Worldwide Influenza Centre<br>London, United Kingdom
A/Norway/2516/2018	A(H3N2)	EPI1255190	EPI_ISL_314674	05.04.2018	WHO National Influenza Centre<br>Oslo, Norway	Crick Worldwide Influenza Centre<br>London, United Kingdom
A/Norway/3275/2018	A(H3N2)	EPI1328875	EPI_ISL_332813	04.10.2018	WHO National Influenza Centre<br>Oslo, Norway	Crick Worldwide Influenza Centre<br>London, United Kingdom
A/La Rioja/2202/2018	A(H3N2)	EPI1256086	EPI_ISL_314925	02.04.2018	Instituto de Salud Carlos III<br>Madrid, Spain	Crick Worldwide Influenza Centre<br>London, United Kingdom
A/Hong Kong/681/2018	A(H3N2)	EPI1336547	EPI_ISL_334100	09.04.2018	Government Virus Unit<br>Kowloon, Hong Kong (SAR)	Crick Worldwide Influenza Centre<br>London, United Kingdom
A/Netherlands/10260/2018	A(H3N2)	EPI1336545	EPI_ISL_334099	15.02.2018	National Institute for Public Health and the Environment (RIVM)<br>Bilthoven, Netherlands	Crick Worldwide Influenza Centre<br>London, United Kingdom
A/England/538/2018	A(H3N2)	EPI1252524	EPI_ISL_314159	26.02.2018	UK Health Security Agency - Colindale<br>London, United Kingdom	Crick Worldwide Influenza Centre<br>London, United Kingdom
B/Malaysia/2506/2004	B/Victoria	EPI175755<br>INSDC: CY038287	EPI_ISL_29398	2004	nicht verfügbar	nicht verfügbar
B/Brisbane/60/2008	B/Victoria	EPI902446	EPI_ISL_246489	04.08.2008	WHO Centre for Reference & Research on Influenza<br>North Melbourne, Australia	Crick Worldwide Influenza Centre<br>London, United Kingdom
B/Paris/1762/2009	B/Victoria	EPI211562	EPI_ISL_60846	09.02.2009	Institut Pasteur<br>Paris, France	National Institute for Medical Research<br>London, United Kingdom
B/Hong Kong/514/2009	B/Victoria	EPI243627	EPI_ISL_70224	11.10.2009	Government Virus Unit<br>Kowloon, Hong Kong (SAR)	National Institute for Medical Research<br>London, United Kingdom

B/Odessa/3886/2010	B/Victoria	EPI902458	EPI_ISL_246495	19.03.2010	Ministry of Health of Ukraine<br>Kiev, Ukraine	Crick Worldwide Influenza Centre<br>London, United Kingdom
B/Malta/MV636714/2011	B/Victoria	EPI346732	EPI_ISL_99941	07.03.2011	Mater Dei Hospital<br>Msida, Malta	National Institute for Medical Research<br>London, United Kingdom
B/Formosa/V2367/2012	B/Victoria	EPI904348	EPI_ISL_246895	06.08.2012	Instituto Nacional de Enfermedades Infecciosas<br>Buenos Aires, Argentina	Crick Worldwide Influenza Centre<br>London, United Kingdom
B/South Australia/81/2012	B/Victoria	EPI566499	EPI_ISL_172974	28.11.2012	Institute of Medical and Veterinary Science<br>Adelaide, Australia	WHO Collaborating Centre for Reference and Research on Influenza<br>Melbourne, Australia
B/Johannesburg/3964/2012	B/Victoria	EPI406272	EPI_ISL_132268	03.08.2012	National Institute for Communicable Disease<br>Johannesburg, South Africa	National Institute for Medical Research<br>London, United Kingdom
B/Nordrhein Westfalen/1/2016	B/Victoria	EPI702285	EPI_ISL_208671	04.01.2016	Robert Koch Institute Nationales Referenzzentrum für Influenza<br>Berlin, Germany	Crick Worldwide Influenza Centre<br>London, United Kingdom
B/Norway/2409/2017	B/Victoria	EPI1015818	EPI_ISL_268030	27.04.2017	WHO National Influenza Centre<br>Oslo, Norway	Crick Worldwide Influenza Centre<br>London, United Kingdom
B/Colorado/06/2017	B/Victoria	EPI1055532	EPI_ISL_277070	25.02.2017	Centers for Disease Control and Prevention<br>Atlanta, United States	Crick Worldwide Influenza Centre<br>London, United Kingdom
B/Hong Kong/269/2017	B/Victoria	EPI1052656	EPI_ISL_276540	24.05.2017	Government Virus Unit<br>Kowloon, Hong Kong (SAR)	Centers for Disease Control and Prevention<br>Atlanta, United States
B/Cote d'Ivoire/1662/2018	B/Victoria	EPI1312316	EPI_ISL_330147	25.07.2018	Pasteur Institut of Côte d'Ivoire<br>Abidjan, Cote d'Ivoire	Crick Worldwide Influenza Centre<br>London, United Kingdom
B/Florida/4/2006	B/Yamagata	EPI159978<br>INSDC:<br>CY033876	EPI_ISL_22808	2006	nicht verfügbar	nicht verfügbar
B/Brisbane/03/2007	B/Yamagata	EPI155964	EPI_ISL_20655	09.03.2007	nicht verfügbar	Centers for Disease Control and Prevention<br>Atlanta, United States
B/Wisconsin/01/2010	B/Yamagata	EPI271545	EPI_ISL_76921	20.02.2010	Wisconsin State Laboratory of Hygiene<br>Madison, United States	Centers for Disease Control and Prevention<br>Atlanta, United States

B/Estonia/55669/2011	B/Yamagata	EPI319345	EPI_ISL_90663	14.03.2011	Health Protection Inspectorate<br>Tallin, Estonia	National Institute for Medical Research<br>London, United Kingdom
B/Stockholm/12/2011	B/Yamagata	EPI346827	EPI_ISL_99990	28.03.2011	Public Health Agency of Sweden<br>Solna, Sweden	National Institute for Medical Research<br>London, United Kingdom
B/Massachusetts/02/2012	B/Yamagata	EPI904336	EPI_ISL_246883	13.03.2012	Centers for Disease Control and Prevention<br>Atlanta, United States	Crick Worldwide Influenza Centre<br>London, United Kingdom
B/Hong Kong/3577/2012	B/Yamagata	EPI392589	EPI_ISL_128126	13.06.2012	Government Virus Unit<br>Kowloon, Hong Kong (SAR)	National Institute for Medical Research<br>London, United Kingdom
B/Novosibirsk/1/2012	B/Yamagata	EPI368752	EPI_ISL_117374	14.02.2012	Russian Academy of Medical Sciences<br>St. Petersburg, Russian Federation	Centers for Disease Control and Prevention<br>Atlanta, United States
B/Phuket/3073/2013	B/Yamagata	EPI540671	EPI_ISL_165881	21.11.2013	WHO Collaborating Centre for Reference and Research on Influenza<br>Melbourne, Australia	National Institute for Medical Research<br>London, United Kingdom
B/Hong Kong/3417/2014	B/Yamagata	EPI543185	EPI_ISL_166764	04.06.2014	Government Virus Unit<br>Kowloon, Hong Kong (SAR)	National Institute for Medical Research<br>London, United Kingdom
B/Mauritius/1791/2017	B/Yamagata	EPI1113662	EPI_ISL_287040	20.09.2017	Central Health Laboratory<br>Candos, Mauritius	Crick Worldwide Influenza Centre<br>London, United Kingdom

INSDC: International Nucleotide Sequence Database Collaboration, NIC: National Influenza Centre, RIVM: Rijksinstituut voor Volksgezondheid en Milieu, SAR: Sonderverwaltungszone, WHO: World Health Organization
